# *rac*-4-Chloro-2-[({2-[(3-chloro-6-hy­droxy-2,4-dimethyl­benz­yl)(meth­yl)amino]­prop­yl}(meth­yl)amino)­meth­yl]-3,5-dimethyl­phenol

**DOI:** 10.1107/S1600536812039694

**Published:** 2012-09-26

**Authors:** Augusto Rivera, Dency José Pacheco, Jaime Ríos-Motta, Mauricio Maldonado, Michael Bolte

**Affiliations:** a Departamento de Química, Facultad de Ciencias, Universidad Nacional de Colombia, Sede Bogotá, Cra 30 No. 45-03, Bogotá, Código Postal 111321, Colombia; b Institut für Anorganische Chemie, J. W. Goethe-Universität Frankfurt, Max-von-Laue-Strasse 7, 60438 Frankfurt/Main, Germany

## Abstract

The title compound, C_23_H_32_Cl_2_N_2_O_2_, a potential chiral ligand for coordination chemistry, was prepared by a two-step reaction. The mol­ecule is located on a crystallographic centre of inversion. As a result, the methyl group bonded to the methyl­ene group is disordered over two equally occupied positions, sharing the same site as the H atom of the chiral C atom. As a further consequence of the crystallographic centrosymmetry, the 1,2-diamino­propane unit adopts an anti­periplanar conformation and the two benzene rings are coplanar. The central chain is in an all-*trans* arrangement. An intra­molecular O—H⋯N hydrogen bond makes an *S*(6) ring motif. A C—H⋯π inter­action links the mol­ecules into one-dimensional chains along the [001] direction.

## Related literature

For the synthesis of the title compound, see: Rivera *et al.* (2010 ▶); Burke (1949 ▶). For the uses of tetra­hydro­salens in coordination chemistry, see: Atwood (1997 ▶). For related structures, see: Rivera *et al.* (2011 ▶); Xu *et al.* (2009 ▶). For reference bond-length data, see: Allen *et al.* (1987 ▶). For graph-set analysis of hydrogen bonds, see: Bernstein *et al.* (1995 ▶).
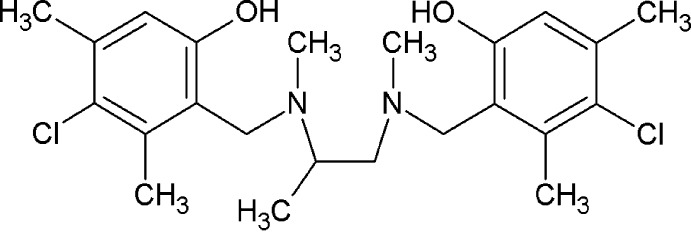


## Experimental

### 

#### Crystal data

C_23_H_32_Cl_2_N_2_O_2_
*M* *_r_* = 439.41
Monoclinic, 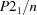

*a* = 9.5011 (8) Å
*b* = 11.9060 (13) Å
*c* = 9.9824 (9) Å
β = 90.348 (7)°
*V* = 1129.19 (19) Å^3^
*Z* = 2
Mo *K*α radiation
μ = 0.31 mm^−1^
*T* = 173 K
0.25 × 0.22 × 0.08 mm

#### Data collection

Stoe IPDS II two-circle diffractometer
Absorption correction: multi-scan (*X-AREA*; Stoe & Cie, 2001 ▶) *T*_min_ = 0.927, *T*_max_ = 0.976
11151 measured reflections
2055 independent reflections
1758 reflections with *I* > 2σ(*I*)
*R*_int_ = 0.074

#### Refinement

*R*[*F* ^2^ > 2σ(*F* ^2^)] = 0.064
*wR*(*F* ^2^) = 0.159
*S* = 1.14
2055 reflections
144 parameters
H atoms treated by a mixture of independent and constrained refinement
Δρ_max_ = 0.59 e Å^−3^
Δρ_min_ = −0.35 e Å^−3^

### 

Data collection: *X-AREA* (Stoe & Cie, 2001 ▶); cell refinement: *X-AREA*; data reduction: *X-AREA*; program(s) used to solve structure: *SHELXS97* (Sheldrick, 2008 ▶); program(s) used to refine structure: *SHELXL97* (Sheldrick, 2008 ▶); molecular graphics: *XP* in *SHELXTL-Plus* (Sheldrick, 2008 ▶); software used to prepare material for publication: *SHELXL97*.

## Supplementary Material

Crystal structure: contains datablock(s) I, global. DOI: 10.1107/S1600536812039694/go2069sup1.cif

Structure factors: contains datablock(s) I. DOI: 10.1107/S1600536812039694/go2069Isup2.hkl

Supplementary material file. DOI: 10.1107/S1600536812039694/go2069Isup3.cml

Additional supplementary materials:  crystallographic information; 3D view; checkCIF report

## Figures and Tables

**Table 1:** Hydrogen-bond geometry (Å, °) *Cg*1 is the centroid of the C11–C16 ring.

*D*—H⋯*A*	*D*—H	H⋯*A*	*D*⋯*A*	*D*—H⋯*A*
O1—H1⋯N1	0.82 (5)	1.87 (5)	2.614 (3)	150 (4)
C1—H1*B*⋯*Cg*1^i^	0.99	2.83	3.709 (3)	148

Symmetry code: (i) 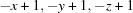.

**Fig. 1.**
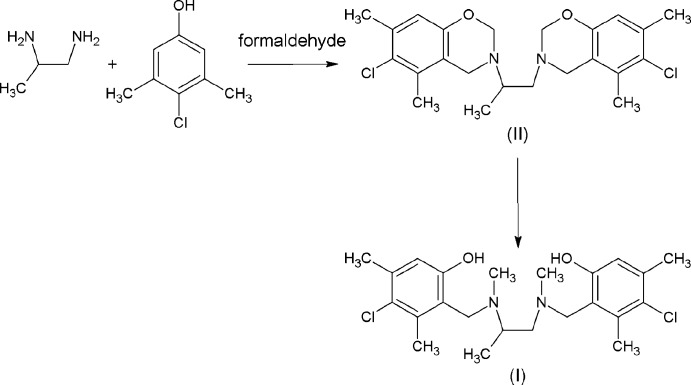
Synthetic route for the title compound.

**Fig. 2.**
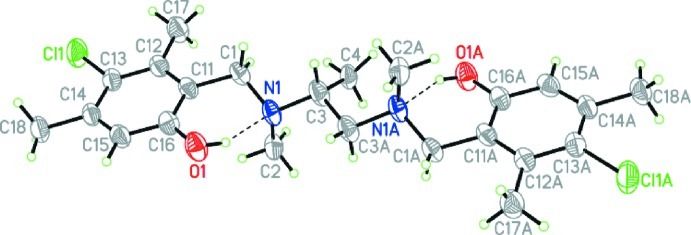
A perspective view of the title compound, showing the atom-numbering scheme. Displacement ellipsoids are drawn at the 50% probability level and H atoms are shown as small spheres of arbitrary radii. Only one of the two positions of the disordered methyl group is shown. Hydrogen bonds are drawn as dashed lines.

## supplementary crystallographic information

### Comment

Compound I, C_23_H_32_Cl_2_N_2_O_2_, a new chiral *N*,*N*-dimethylated tetrahydrosalen (H2[H4]salen) was obtained by reacting the bis-benzoxazine (II) with sodium borohydride using our procedure reported earlier (Rivera *et al.* 2010). The intermediate II was prepared by condensing 1,2-diaminepropane with formaldehyde and 4-chloro-3,5-dimethylphenol employing the general procedure of Burke (1949). The synthetic route for the title compound reported herein is illustrated in Fig. 1. The molecular structure and atom-numbering scheme for title compound, C_23_H_32_Cl_2_N_2_O_2_, are shown in Fig. 2. The bond/newcifs lengths (Allen *et al.*, 1987) and angles are normal and similar to those observed for related structures (Rivera *et al.* 2010; Xu *et al.* 2009). In the title molecule, the 1,2-propanodiamine unit adopts an antiperiplanar conformation with an N1—C3—C3a—N1a torsion angle of -180.0 (2)°. As a consequence of this conformation, both benzene rings are parallel to each other. The central chain –CH_2_—N(CH_3_)—CH_2_—CH(CH_3_)—N(CH_3_)—CH_2_– is found in an all-*trans* arrangement. The two symmetry-related methyl substituents in the molecule (C2 and C2^i^, (i) = 1 - *x*, 1 - *y*, 2 - *z*) are orientated in an antiperiplanar arrangement (pseudo torsion angle CH_3_—N···N—CH_3_ = 180.00°). The C2 and C4 methyl groups are almost (+)-synclinal [C2—N1—C3—C4 torsion angle = 48.0 (4)°], a conformation stabilized by an intramolecular O—H···N hydrogen bond. The relationship of C4 methyl to C2^i^ is defined by the pseudo torsion angle C4—C3···N1a—C2a, which is 114.64 (3)°.

The intramolecular O—H···N hydrogen bond (Table 1) makes an S(6) ring motif (Bernstein *et al.*, 1995), contrasting with the related structure (Xu *et al.*, 2009). In the crystal structure, intermolecular C1—H1B···*Cg1*(1 - *x*, 1 - *y*, 1 - *z*) interaction links the molecules into one-dimensional chains. C1···*Cg1* is 3.709 (3)Å, H1···*Cg1* is 2.83Å and the angle at H1 is 148°. *Cg1* is the centroid of the C11–C16 ring.

### Experimental

Sodium borohydride (3.0 mmol, 0.11 g) was added to a solution of 3,3'-(propane-1,2-diyl)bis(6-chloro-5,7-dimethyl-3,4-dihydro-2 h-benzo[*e*][1,3]oxazina) (435 mg, 1 mmol) (II) in ethanol (15 ml), and the mixture was stirred magnetically for 30 min at room temperature. After completion of the reaction, the mixture was poured into ice-cold water, neutralized with ammonium chloride (12 ml), and extracted with CHCl_3_ (3 × 10 ml). The combined extracts were dried over anhydrous Na_2_SO_4_ and evaporated. Recrystallization from ethanol afforded (I) in 91% yield. m.p. 424 K.

### Refinement

All H atoms bonded to C were refined using a riding model with fixed individual displacement parameters [*U*_iso_(H) = 1.2*U*_eq_(C) or *U*_iso_(H) = 1.5*U*_eq_(C_methyl_] with C—H ranging from 0.95 Å to 0.99 Å. The hydroxyl H atom was isotropically refined. The methyl group C4 is disordered over two centrosymmerically related positions each with a 50% occupancy. The completeness of the data is 99.4% with eleven reflections missing for a full completeness. Since no reflection was omitted on purpose, this is most probably due to the data collection strategy using an area detector.

### Crystal data

**Table:**

C_23_H_32_Cl_2_N_2_O_2_	*F*(000) = 468
*M**_r_* = 439.41	*D*_x_ = 1.292 Mg m^−^^3^
Monoclinic, *P*2_1_/*n*	Mo *K*α radiation, λ = 0.71073 Å
Hall symbol: -P 2yn	Cell parameters from 11821 reflections
*a* = 9.5011 (8) Å	θ = 2.4–28.1°
*b* = 11.9060 (13) Å	µ = 0.31 mm^−^^1^
*c* = 9.9824 (9) Å	*T* = 173 K
β = 90.348 (7)°	Plate, colourless
*V* = 1129.19 (19) Å^3^	0.25 × 0.22 × 0.08 mm
*Z* = 2	

### Data collection

**Table:**

Stoe IPDS II two-circle diffractometer	2055 independent reflections
Radiation source: Genix 3D IµS microfocus X-ray source	1758 reflections with *I* > 2σ(*I*)
Genix 3D multilayer optics monochromator	*R*_int_ = 0.074
ω scans	θ_max_ = 25.3°, θ_min_ = 3.4°
Absorption correction: multi-scan (*X-AREA*; Stoe & Cie, 2001)	*h* = −11→11
*T*_min_ = 0.927, *T*_max_ = 0.976	*k* = −14→14
11151 measured reflections	*l* = −11→11

### Refinement

**Table:**

Refinement on *F*^2^	Primary atom site location: structure-invariant direct methods
Least-squares matrix: full	Secondary atom site location: difference Fourier map
*R*[*F*^2^ > 2σ(*F*^2^)] = 0.064	Hydrogen site location: inferred from neighbouring sites
*wR*(*F*^2^) = 0.159	H atoms treated by a mixture of independent and constrained refinement
*S* = 1.14	*w* = 1/[σ^2^(*F*_o_^2^) + (0.0629*P*)^2^ + 0.952*P*] where *P* = (*F*_o_^2^ + 2*F*_c_^2^)/3
2055 reflections	(Δ/σ)_max_ < 0.001
144 parameters	Δρ_max_ = 0.59 e Å^−^^3^
0 restraints	Δρ_min_ = −0.35 e Å^−^^3^

### Special details

**Table:**

Experimental. 1H NMR (400.1 MHz, CDCl_3_): δ 1.07 (d, ^3^J = 6.6 Hz, 3H), 2.18 (s, 3H),2.25 (s, 3H), 2.30 (s, 6H), 2.31 (s, 6H), 2.35 (dd, ^2^Jgem = 12.6, ^3^J = 6.9 Hz, 1H), 2.62 (dd, ^2^Jgem = 12.6, ^3^J = 6.7 Hz, 1H), 3.03–3.11 (m, 1H), 3.69 (d, ^2^Jgem = 14.0, 1H), 3.74 (d, ^2^Jgem = 14.0, 1H), 3.81 (s, 2H), 6.62 (s, 2H) ^13^C NMR (100.6 MHz, CDCl_3_): δ 11.17, 16.74, 16.82, 21.22, 21.24, 35.19, 41.9, 53.68, 54.20, 58.57, 60.32, 116.57, 116.58, 118.48, 118.94, 125.26, 125.39, 133.96, 134.04, 136.62, 136.73, 156.76, 156.78. F T—IR (KBr) (ν, cm^-1^): 3406 (O—H, broad, m), 2960 (CH_3_*asym, st*), 2922 (CH_2_*asym*, st), 2855 (CH_3_*sym,* st), 2802 (CH_2_*sym*, st), 1613 (–C=C, st), 1320 (C—N, st), 668 (C—Cl, st).
Geometry. All e.s.d.'s (except the e.s.d. in the dihedral angle between two l.s. planes) are estimated using the full covariance matrix. The cell e.s.d.'s are taken into account individually in the estimation of e.s.d.'s in distances, angles and torsion angles; correlations between e.s.d.'s in cell parameters are only used when they are defined by crystal symmetry. An approximate (isotropic) treatment of cell e.s.d.'s is used for estimating e.s.d.'s involving l.s. planes.
Refinement. Refinement of *F*^2^ against ALL reflections. The weighted *R*-factor *wR* and goodness of fit *S* are based on *F*^2^, conventional *R*-factors *R* are based on *F*, with *F* set to zero for negative *F*^2^. The threshold expression of *F*^2^ > σ(*F*^2^) is used only for calculating *R*-factors(gt) *etc*. and is not relevant to the choice of reflections for refinement. *R*-factors based on *F*^2^ are statistically about twice as large as those based on *F*, and *R*-factors based on ALL data will be even larger.

### Fractional atomic coordinates and isotropic or equivalent isotropic displacement parameters (Å^2^)

**Table:**

	*x*	*y*	*z*	*U*_iso_*/*U*_eq_	Occ. (<1)
Cl1	0.74219 (10)	0.71021 (8)	0.25120 (9)	0.0590 (3)	
N1	0.5987 (3)	0.54354 (18)	0.8459 (2)	0.0383 (6)	
O1	0.7205 (2)	0.39166 (17)	0.6972 (3)	0.0485 (6)	
H1	0.687 (5)	0.421 (4)	0.764 (5)	0.071 (13)*	
C1	0.5450 (3)	0.5882 (3)	0.7189 (3)	0.0410 (7)	
H1A	0.5303	0.6702	0.7282	0.049*	
H1B	0.4525	0.5535	0.6992	0.049*	
C2	0.7218 (4)	0.6084 (3)	0.8905 (3)	0.0479 (8)	
H2A	0.7945	0.6057	0.8214	0.072*	
H2B	0.7588	0.5762	0.9739	0.072*	
H2C	0.6940	0.6866	0.9059	0.072*	
C3	0.4831 (3)	0.5398 (2)	0.9448 (3)	0.0464 (8)	
H3	0.3984	0.5110	0.8981	0.056*	
H3'	0.4654	0.6157	0.9814	0.056*	0.50
C4	0.4443 (7)	0.6608 (5)	1.0048 (6)	0.0403 (13)	0.50
H4A	0.5114	0.6802	1.0760	0.060*	0.50
H4B	0.3489	0.6588	1.0416	0.060*	0.50
H4C	0.4489	0.7172	0.9335	0.060*	0.50
C11	0.6426 (3)	0.5669 (2)	0.6023 (3)	0.0346 (6)	
C12	0.6469 (3)	0.6424 (2)	0.4950 (3)	0.0370 (6)	
C13	0.7364 (3)	0.6175 (2)	0.3882 (3)	0.0386 (7)	
C14	0.8205 (3)	0.5226 (2)	0.3842 (3)	0.0372 (7)	
C15	0.8119 (3)	0.4494 (2)	0.4901 (3)	0.0411 (7)	
H15	0.8676	0.3832	0.4896	0.049*	
C16	0.7241 (3)	0.4695 (2)	0.5979 (3)	0.0371 (6)	
C17	0.5559 (4)	0.7462 (3)	0.4936 (4)	0.0539 (9)	
H17A	0.6010	0.8052	0.5473	0.081*	
H17B	0.4636	0.7284	0.5314	0.081*	
H17C	0.5440	0.7725	0.4012	0.081*	
C18	0.9182 (3)	0.4993 (3)	0.2693 (3)	0.0506 (8)	
H18A	0.9715	0.4305	0.2874	0.076*	
H18B	0.9836	0.5624	0.2590	0.076*	
H18C	0.8632	0.4900	0.1867	0.076*	

### Atomic displacement parameters (Å^2^)

**Table:**

	*U*^11^	*U*^22^	*U*^33^	*U*^12^	*U*^13^	*U*^23^
Cl1	0.0693 (6)	0.0651 (6)	0.0429 (5)	0.0039 (4)	0.0184 (4)	0.0072 (4)
N1	0.0397 (13)	0.0333 (12)	0.0421 (14)	−0.0020 (9)	0.0198 (11)	0.0028 (10)
O1	0.0586 (14)	0.0358 (11)	0.0515 (14)	0.0070 (9)	0.0185 (11)	0.0011 (10)
C1	0.0323 (15)	0.0451 (16)	0.0457 (18)	0.0006 (12)	0.0128 (12)	0.0010 (13)
C2	0.0512 (19)	0.0565 (18)	0.0361 (17)	−0.0031 (15)	0.0128 (14)	−0.0041 (14)
C3	0.0454 (18)	0.0442 (16)	0.0499 (19)	0.0062 (13)	0.0217 (14)	0.0130 (14)
C4	0.049 (3)	0.040 (3)	0.032 (3)	0.007 (2)	0.015 (3)	0.008 (2)
C11	0.0263 (13)	0.0379 (14)	0.0396 (16)	−0.0048 (10)	0.0067 (11)	−0.0064 (11)
C12	0.0332 (14)	0.0396 (15)	0.0383 (16)	−0.0025 (11)	0.0055 (12)	−0.0041 (12)
C13	0.0372 (15)	0.0436 (15)	0.0350 (16)	−0.0044 (12)	0.0060 (12)	−0.0047 (12)
C14	0.0300 (14)	0.0469 (16)	0.0348 (15)	−0.0060 (12)	0.0059 (11)	−0.0117 (12)
C15	0.0362 (15)	0.0422 (15)	0.0448 (17)	0.0034 (12)	0.0040 (13)	−0.0134 (13)
C16	0.0359 (15)	0.0342 (14)	0.0413 (16)	−0.0045 (11)	0.0069 (12)	−0.0036 (12)
C17	0.061 (2)	0.0508 (18)	0.050 (2)	0.0165 (15)	0.0152 (16)	0.0071 (15)
C18	0.0440 (17)	0.067 (2)	0.0415 (18)	0.0017 (15)	0.0125 (14)	−0.0143 (15)

### Geometric parameters (Å, º)

**Table:**

Cl1—C13	1.758 (3)	C4—H4B	0.9800
N1—C1	1.464 (4)	C4—H4C	0.9800
N1—C2	1.468 (4)	C11—C16	1.396 (4)
N1—C3	1.483 (4)	C11—C12	1.399 (4)
O1—C16	1.358 (4)	C12—C13	1.400 (4)
O1—H1	0.82 (5)	C12—C17	1.508 (4)
C1—C11	1.514 (4)	C13—C14	1.385 (4)
C1—H1A	0.9900	C14—C15	1.373 (4)
C1—H1B	0.9900	C14—C18	1.506 (4)
C2—H2A	0.9800	C15—C16	1.386 (4)
C2—H2B	0.9800	C15—H15	0.9500
C2—H2C	0.9800	C17—H17A	0.9800
C3—C3^i^	1.486 (6)	C17—H17B	0.9800
C3—C4	1.604 (6)	C17—H17C	0.9800
C3—H3	0.9886	C18—H18A	0.9800
C3—H3'	0.9905	C18—H18B	0.9800
C4—H3'	0.6186	C18—H18C	0.9800
C4—H4A	0.9800		
			
C1—N1—C2	110.1 (2)	C16—C11—C12	119.5 (3)
C1—N1—C3	109.4 (2)	C16—C11—C1	120.4 (3)
C2—N1—C3	113.9 (2)	C12—C11—C1	120.1 (2)
C16—O1—H1	108 (3)	C11—C12—C13	117.9 (3)
N1—C1—C11	113.1 (2)	C11—C12—C17	121.0 (3)
N1—C1—H1A	108.9	C13—C12—C17	121.1 (3)
C11—C1—H1A	108.9	C14—C13—C12	123.3 (3)
N1—C1—H1B	108.9	C14—C13—Cl1	117.9 (2)
C11—C1—H1B	108.9	C12—C13—Cl1	118.8 (2)
H1A—C1—H1B	107.8	C15—C14—C13	117.3 (3)
N1—C2—H2A	109.5	C15—C14—C18	120.7 (3)
N1—C2—H2B	109.5	C13—C14—C18	122.1 (3)
H2A—C2—H2B	109.5	C14—C15—C16	121.9 (3)
N1—C2—H2C	109.5	C14—C15—H15	119.1
H2A—C2—H2C	109.5	C16—C15—H15	119.1
H2B—C2—H2C	109.5	O1—C16—C15	117.9 (3)
N1—C3—C3^i^	110.8 (3)	O1—C16—C11	121.9 (3)
N1—C3—C4	113.2 (3)	C15—C16—C11	120.3 (3)
C3^i^—C3—C4	110.2 (4)	C12—C17—H17A	109.5
N1—C3—H3	107.5	C12—C17—H17B	109.5
C3^i^—C3—H3	107.5	H17A—C17—H17B	109.5
C4—C3—H3	107.4	C12—C17—H17C	109.5
N1—C3—H3'	110.3	H17A—C17—H17C	109.5
C3^i^—C3—H3'	110.1	H17B—C17—H17C	109.5
H3—C3—H3'	110.5	C14—C18—H18A	109.5
C3—C4—H4A	109.5	C14—C18—H18B	109.5
C3—C4—H4B	109.5	H18A—C18—H18B	109.5
H4A—C4—H4B	109.5	C14—C18—H18C	109.5
C3—C4—H4C	109.5	H18A—C18—H18C	109.5
H4A—C4—H4C	109.5	H18B—C18—H18C	109.5
H4B—C4—H4C	109.5		
			
C2—N1—C1—C11	69.4 (3)	C11—C12—C13—Cl1	179.3 (2)
C3—N1—C1—C11	−164.7 (2)	C17—C12—C13—Cl1	0.3 (4)
C1—N1—C3—C3^i^	159.9 (3)	C12—C13—C14—C15	1.2 (4)
C2—N1—C3—C3^i^	−76.3 (4)	Cl1—C13—C14—C15	−178.2 (2)
C1—N1—C3—C4	−75.7 (4)	C12—C13—C14—C18	−178.6 (3)
C2—N1—C3—C4	48.0 (4)	Cl1—C13—C14—C18	2.0 (4)
N1—C1—C11—C16	33.1 (4)	C13—C14—C15—C16	−0.7 (4)
N1—C1—C11—C12	−149.8 (3)	C18—C14—C15—C16	179.1 (3)
N1—C3—C3^i^—N1^i^	−180.0 (2)	C14—C15—C16—O1	178.9 (3)
C16—C11—C12—C13	−1.4 (4)	C14—C15—C16—C11	−0.9 (4)
C1—C11—C12—C13	−178.5 (3)	C12—C11—C16—O1	−177.8 (3)
C16—C11—C12—C17	177.5 (3)	C1—C11—C16—O1	−0.7 (4)
C1—C11—C12—C17	0.4 (4)	C12—C11—C16—C15	2.0 (4)
C11—C12—C13—C14	−0.2 (4)	C1—C11—C16—C15	179.1 (3)
C17—C12—C13—C14	−179.1 (3)		

Symmetry code: (i) −*x*+1, −*y*+1, −*z*+2.

### Hydrogen-bond geometry (Å, º)

**Table:**

*D*—H···*A*	*D*—H	H···*A*	*D*···*A*	*D*—H···*A*
O1—H1···N1	0.82 (5)	1.87 (5)	2.614 (3)	150 (4)
C1—H1*B*···*Cg*1^ii^	0.99	2.83	3.709 (3)	148

Symmetry code: (ii) −*x*+1, −*y*+1, −*z*+1.
